# Integrating sustainability in dentistry: a pathway towards achieving the UN 2030 agenda

**DOI:** 10.3389/froh.2025.1549020

**Published:** 2025-07-11

**Authors:** Matilde Guerra, Mariana Morgado, Yago Leira, Tiago Leitão, João Botelho, José João Mendes

**Affiliations:** ^1^ Egas Moniz Center for Interdisciplinary Research (CiiEM), Egas Moniz School of Health & Science, Caparica, Almada, Portugal; ^2^ Periodontology Unit, Faculty of Odontology and Medicine, University of Santiago de Compostela, Santiago de Compostela, Spain

**Keywords:** environmental impact, dentistry, sustainability, sustainable development, dental materials

## Abstract

The growing impact of the climate crisis has raised significant concerns due to its profound effects on public health and ecological stability. This article explores how the implementation of sustainable practices in dentistry can contribute to a more efficient, safe and environmentally responsible approach, in line with the Sustainable Development Goals outlined in the United Nations’ 2030 Agenda. A key focus is on waste reduction, which is achieved through the promotion of oral health and disease prevention, thereby minimising the waste and carbon emissions associated with dental care. In addition, the responsible use of resources such as water and energy and the use of environmentally sustainable dental materials play a critical role in reducing the environmental footprint of dentistry. Key measures such as the recycling of single-use plastics are essential to curb pollution and reduce the uptake of microplastics by marine ecosystems and humans. Education and awareness of sustainability is also essential. This can be promoted by integrating sustainable principles into the curriculum of dental courses and within scientific committees. Promoting these practices among emerging health professionals and researchers will drive the development of innovative alternatives that further reduce the environmental impact of the field.

## 1 Introduction

The issue of climate change continues to be a topic of considerable debate, driven by the increasing global impact of greenhouse gas emissions, which have reached unprecedented levels in human history (1). These emissions result in the trapping of heat within the atmosphere, which in turn causes a rise in global temperatures. Since the advent of the Industrial Revolution, the global mean temperature has increased by approximately 1.1°C, with CO_2_ emissions contributing to a 25% acidification of oceanic pH relative to pre-industrial levels (2). Such changes have resulted in alterations to climate patterns, accompanied by an increase in sea levels and the occurrence of extreme weather events, including fires, droughts, heat waves and floods (1). These developments present considerable risks to human health and well-being.

The ramifications of these alterations on human health implications are extensive and far-reaching. Elevated CO_2_ levels have been linked to a range of physiological issues, including inflammation, respiratory acidosis and psychological changes (2). Air pollution is responsible for approximately seven million deaths annually, with associated diseases including lung cancer and cardiovascular disease. Furthermore, extreme temperatures and exposure to pollutants from fires have been linked to an exacerbation of respiratory infections, developmental disorders and chronic diseases such as asthma (2). Furthermore, flooding can result in trauma, infection, and long-term health complications related to waterborne pathogens (2).

In order to address these challenges, the international community has adopted a series of key frameworks with the objective of mitigating climate change and promoting sustainability. The Paris Agreement, established at COP21 in 2015, set a global goal to limit the increase in global temperatures to below 2°C, with an optimal objective of limiting the increase to 1.5°C (3). Moreover, the 2030 Agenda for Sustainable Development, adopted by the United Nations in 2015, introduced 17 Sustainable Development Goals (SDGs), which encompass environmental, economic and social targets to be achieved by 2030 (4).

Given that the healthcare sector, which is responsible for approximately 5% of global CO_2_ emissions (5), it is imperative that efforts are made to improve environmental sustainability are paramount. Dentistry, as a significant component of this sector, has the potential to play an instrumental role in reducing its environmental footprint. In response to the 2030 Agenda, the Fédération Dentaire Internationale (FDI), in collaboration with the University of Sheffield, has formulated the “Consensus on Environmentally Sustainable Oral Healthcare: A Joint Stakeholder Statement” (6). The Consensus identifies sustainable dental practices, with a particular focus on waste reduction, resource efficiency and oral health promotion as strategies for reducing the sector's overall environmental impact of the sector.

FDI's sustainability framework is based on the four 4Rs of reduce, reuse, recycle and rethink, with a focus on preventive, operational and integrated care and an overarching emphasis on ownership of care (6). These strategies are aligned with several UN SDGs, including those related to health, education, clean water, responsible consumption, and climate action.

The objective of this literature review is to examine the environmental sustainability strategies pertinent to the field of dentistry, with a specific emphasis on materials production, waste management and clinical patient care. In alignment with the UN 2030 Agenda and the FDI Consensus, this review addresses pivotal strategies for aligning dentistry with global sustainability goals and elucidates the sector's role in advancing a more environmentally conscious healthcare system.

## 2 Methodology

This review adopted a structured approach, commencing with the establishment of the eligibility criteria. The majority of the included articles were published within the last decade, with the exception of a single review from 2007 that focused on mercury from dental amalgams. In light of the growing emphasis on sustainability in recent years, priority was given to recent studies over older investigations. Only reviews published in English and Portuguese were considered. Legislation specific to individual countries was deemed irrelevant to this review.

A comprehensive literature search was conducted across multiple databases, including PubMed and Elsevier, with Google Scholar serving as an additional resource for topics outside the health sector. Relevant information was also gathered from authoritative sources such as the United Nations, the World Health Organization, and the European Union.

A combination of Medical Subject Headings (MeSH) and free text keywords were used. Boolean operators (AND/OR) and truncation were used to expand or refine the search as appropriate. The search strategy was tailored to cover several thematic areas relevant to environmental sustainability in dentistry. A summary of the search terms by topic is given in Table 1.

**Table 1:** Structured search strategy with thematic search terms used in the narrative review.

Topic area	Search terms used
Dental amalgam & mercury pollution	“dental amalgam” AND (mercury OR MeHg OR “wastewater” OR “effluent” OR “pollution” OR “cremation” OR “bioaccumulation” OR “Minamata disease”)
Heavy metals in dentistry	“dentistry” AND (“heavy metals” OR “mercury” OR “lead” OR “cadmium” OR “copper” OR “zinc”) AND (“occupational exposure” OR “environmental contamination”)
Resin-based composite waste	“resin-based composite” OR “RBC” AND (“monomer elution” OR “degradation” OR “microparticles” OR “nanoplastics” OR “BisGMA” OR “TEGDMA” OR “HEMA” OR “UDMA”)
Composite manufacturing pollution	“composite materials” AND (“formaldehyde” OR “styrene” OR “volatile organic compounds” OR “HAPs” OR “manufacturing emissions”)
Biocomposites and sustainable materials	“biocomposites” OR “hybrid composites” AND (“natural fibers” OR “renewable resources” OR “NFPC” OR “agricultural waste” OR “sustainable materials”)
CAD/CAM and indirect restorations	“CAD/CAM” AND (“subtractive manufacturing” OR “environmental impact” OR “material waste” OR “dental milling” OR “microplastics”)
Occupational exposure & health risks	“dental clinic” AND (“occupational exposure” OR “aerosols” OR “airborne particles” OR “dermal exposure” OR “lung disease” OR “skin disorders”)
Carbon footprint of dentistry	“carbon footprint” AND (“dental practice” OR “patient travel” OR “appointment frequency” OR “green dentistry” OR “clinical efficiency”)
Cremation & dental material emissions	“dental materials” AND “cremation” AND (“mercury release” OR “pollution” OR “composite particles” OR “environmental impact”)
Environmental lifecycle of dental products	“life cycle assessment” OR “LCA” AND “dental materials” OR “sustainability in dentistry”
Circular economy in healthcare	“circular economy” AND (“dentistry” OR “healthcare” OR “waste reduction” OR “recycling” OR “material reuse”)
Integrated and sustainable care models	“integrated care” AND “dentistry” AND (“sustainable practice” OR “clinical leadership” OR “team-based care” OR “value-based care”)
Alignment with SDGs	“sustainable development goals” OR “SDGs” AND (“oral health” OR “dental sustainability” OR “environmental health” OR “climate action”)

**Figure 1:**
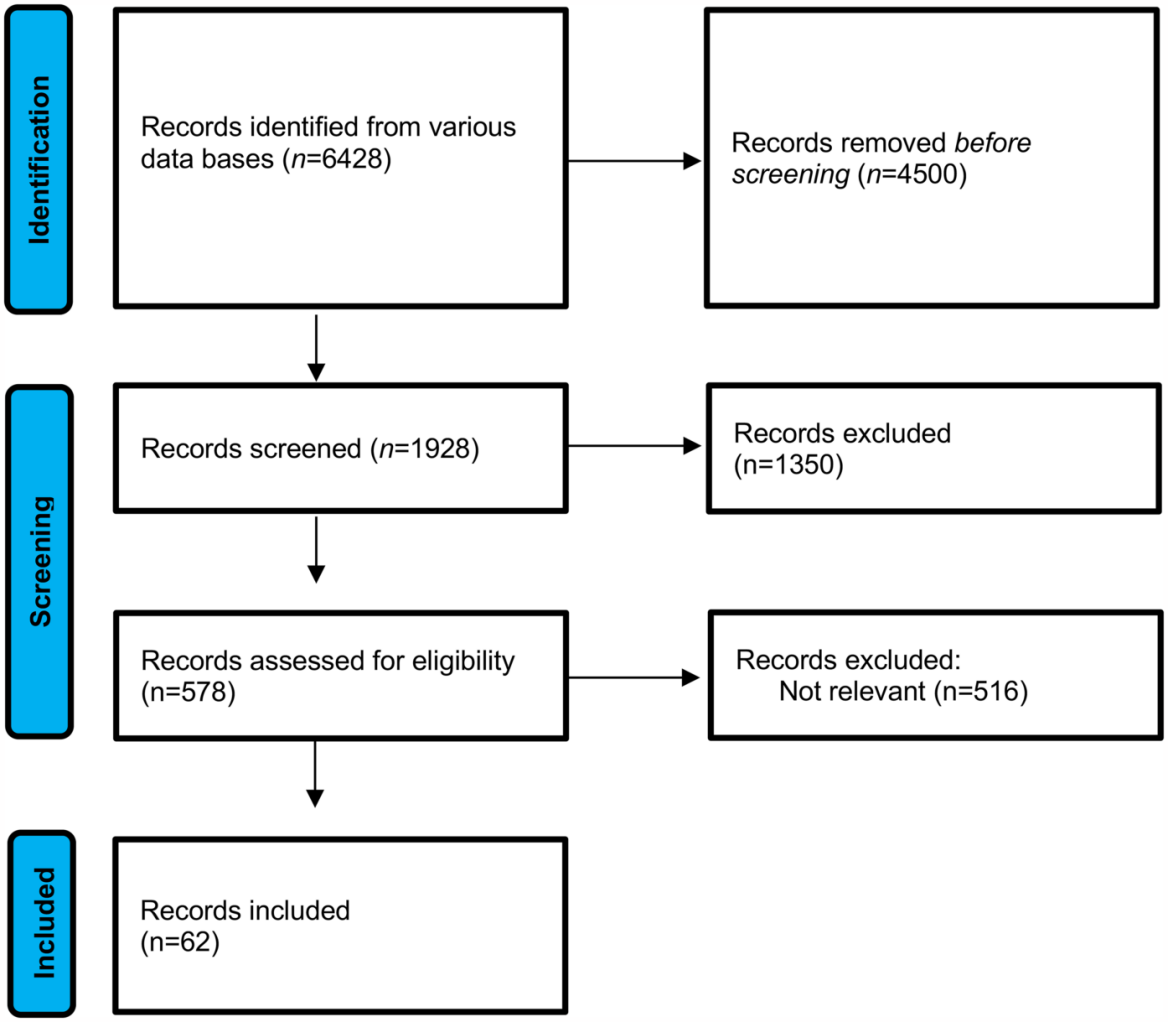
PRISMA flow diagram illustrating the study selection process (7).

The inclusion and exclusion criteria applied in the selection process are illustrated in the PRISMA flow diagram (Figure 1).

## 3 The environmental impact of dentistry

The environmental impact of human activities can be evaluated using the ecological footprint, a metric developed by the Global Footprint Network for the purpose of assessing resource consumption and waste generation in relation to the capacity of the natural environment to absorb waste and replenish resources (8). In the context of healthcare, particularly in dental medicine, in the field of dentistry, several key factors contribute to this environmental burden.

### 3.1 Patient and staff transportation

A study conducted in England between 2013 and 2014 evaluated the carbon footprint of National Health Service (NHS) oral health services. The findings indicated that both patient and staff travel were identified as significant contributors to emissions. In particular, emissions resulting from patient travel constituted 31.1% of the total, while emissions from staff commuting accounted for 30.3% and emissions from business travel for 3.1%. This equates to approximately 435 kt of CO₂ equivalent (ktCO₂e) per year (9). In order to offset these emissions, it would be necessary to plant over 28 million trees across 16,835 hectares (10).

In addition to CO_2_, transport-related activities release pollutants such as nitrogen oxides (NOx) and particulate matter (PM2.5), which contribute to the loss of 325 quality-adjusted life years (QALYs) per year and an economic burden of £17.5 million in health and society (10).

### 3.2 Single-use plastics

The healthcare sector, including dentistry, represents a significant contributor to plastic waste, accounting for 53% of the total plastic waste generated by the NHS. Between 2016 and 2017, the NHS generated 590,000 t of plastic waste, a figure that is predicted to increase to 6.3 million tonnes by 2030 (11). Single-use plastics (SUPs), including items such as gloves, masks and sterilisation sleeves, are commonly utilised in dental procedures. Gloves represent the most common form of single-use plastic (SUP) waste, with an average of 352 million discarded annually in the UK alone (11).

A study found that an average number of 21 SUP, with a mean mass of 354 g *per* procedure (including setup and cleanup), are used per dental, resulting to the generation of 14.4 t of SUP waste annually (11). While direct restorations generate the highest volume of SUP waste *per* procedure, dental examinations contribute a larger overall volume due to their higher frequency (11).

### 3.3 Pollution from heavy metal residues

The presence of heavy metals, including silver (Ag) and lead (Pb), in dental materials such as x-ray films and protective aprons, raises concerns regarding environmental risk if improper disposal practices are employed (12, 13). Of greater concern is the use of dental amalgam, which contains approximately 50% mercury (Hg). In 2018, approximately 75 t of dental amalgam were used annually in the EU (14). The improper disposal of this material has the potential to result in significant environmental contamination.

Mercury from dental amalgam can enter the environment via several pathways, including the release of unused amalgam particles, particles generated during the carving or removal of fillings, and particles shed by patients. Additionally, amalgam particles may enter wastewater from dental clinics or be released during cremation. The quantity of mercury released during cremation is estimated to be between 0.25 and 1 gram per cremation (12, 14). Once released, mercury can be methylated by bacteria to form the toxic methylmercury (MeHg), which has the capacity to bioaccumulate in aquatic food chains. The ingestion of fish contaminated with mercury can result in significant health risks to humans, affecting multiple organ systems and leading to oral health complications such as osteitis, gingivitis and ulcerative lesions (13, 14). In 2008, a study was published on Minamata disease, which refers to the poisoning that occurred in 1956 as a result of humans consuming fish and shellfish contaminated with methylmercury (MeHg) released from a chemical plant. The study identified 2,252 patients with Minamata disease, of whom 1,043 had died (15).

Despite the advances in waste disposal methods, the ultimate environmental fate of dental mercury remains inadequately defined, with significant amounts potentially entering the environment due to historical poor practices (16). In addition to mercury, wastewater from dental practices has been shown to contain high concentrations of other heavy metals such as copper and zinc, often exceeding acceptable environmental standards (17). These contaminants, including metallic nanoparticles, pose not only environmental risks but also occupational hazards for dental professionals and patients (13). Chronic exposure to these metals in clinical settings has been associated with dermatological and respiratory conditions among dental staff (18). Furthermore, human teeth dentine has emerged as a reliable bio-indicator for assessing long-term exposure to environmental metal pollution, with metal concentrations increasing with age and varying by gender and ethnicity (18).

To mitigate these environmental and health risks, the installation of amalgam separators in dental clinics is recommended to capture mercury before it enters wastewater systems (16). However, the effectiveness of these separators can vary, and they may not capture all forms of mercury, particularly dissolved mercury (16). Therefore, additional treatment methods, such as advanced filtration systems or chemical treatments, may be necessary to further reduce mercury levels in dental wastewater (16). Emerging nanotechnologies also offer promising solutions for environmental remediation, though their application beyond laboratory settings requires further investigation (19).

### 3.4 Pollution from resin-based composite waste

Resin-based composites (RBCs) are a prevalent alternative to dental amalgam for direct restorations. Nevertheless, in contrast with the prevailing view, RBCs also present a risk to human health and the environment.

A significant concern is the potential toxicity of resin monomers, such as bisphenol A-glycidyl methacrylate (Bis-GMA), which contains bisphenol A (BPA), and triethylene glycol dimethacrylate (TEGDMA). In dental procedures utilising composite resins, only 60%–75% of the monomers are converted into polymers, with a proportion of unreacted monomers being released into the environment and contributing to pollution (14).

The pollution generated by RBCs commences during the manufacturing process. Waste generated, including expired or excess resins, is frequently deposited in landfills without undergoing polymerisation, thereby releasing harmful substances into the environment (14). Recent research has demonstrated that the grinding of resin-based composites can lead to the release of bisphenol A (BPA) into dental wastewater, with concentrations varying according to the type of composite material used. The study also highlighted the effectiveness of catalytic carbon filtration systems in significantly reducing BPA levels, suggesting a viable mitigation strategy to prevent environmental contamination (20). The use of subtractive methods in indirect restorations via CAD/CAM technology has been identified as a source of environmental contamination, with the generation of microparticles and microplastics in the water used during the process (21). Furthermore, the cremation of individuals with RBC restorations results in the release of these particles into the atmosphere or waterways (14, 21).

Research has indicated that RBC dental materials have the capacity to release a variety of chemical compounds into the environment, thereby posing a potential threat of pollution. These materials have been observed to elute monomers and microparticles through degradation processes, with the majority of leaching occurring within hours of placement (21, 22). The extent of elution is contingent on factors such as the degree of cure, the solvent composition, and the characteristics of the particles (21, 23). RBC microparticles possess substantial surface areas, enabling sustained elution of constituents over extended periods (23). While monomers such as HEMA, TEGDMA, BisGMA, and UDMA are the focus of many studies, additives such as initiators and stabilisers may also be released in significant quantities (22). The environmental impact of these pollutants on biodiversity remains to be elucidated, necessitating further research and the implementation of mitigation strategies throughout the RBC lifecycle (23).

Moreover, the production of composite materials is linked to the emission of hazardous air pollutants, such as styrene and formaldehyde, which have been demonstrated to possess deleterious effects on human health (24). In response to these concerns, there is an increasing interest in the development of biocomposites and hybrid composites using renewable and recycled resources, particularly in industries such as automotive manufacturing (25). Natural fibre-polymer composites (NFPCs) derived from agricultural and forest industry waste have been identified as a promising, eco-friendly alternative to petroleum-based materials (26). These materials not only provide an alternative to traditional composites but also present a higher-value option for sustainable waste management. Nevertheless, challenges persist in enhancing material compatibility and performance, underscoring the necessity for additional research to support sustainability objectives in composite manufacturing (25).

A comparative life cycle assessment of dental restorative materials revealed that composite-based materials, while popular, present significant environmental impacts, particularly in production and disposal (27). This assessment highlights the importance of developing more sustainable alternatives in dental material design and encourages further innovations to reduce these negative effects.

Additionally, recent studies have also indicated that microparticles released from RBCs may cause additional environmental damage, affecting aquatic ecosystems and potentially leading to the bioaccumulation of these toxic compounds (28). Life cycle analyses of the environmental impact of dental practices, including waste generation and water use, demonstrate the urgent need for sustainable solutions within the dental industry (29). Implementing sustainable practices in procurement, waste management, and water usage within dental clinics can substantially reduce the overall carbon footprint, as evidenced by life cycle analyses in dental settings (29).

### 3.5 Carbon footprint of dental procedures

In evaluating the carbon footprint of dental procedures, two key factors must be taken into account: the individual carbon footprint of each procedure and the cumulative footprint based on the frequency of procedures (30).

It is notable that procedures requiring multiple visits, such as fixed and removable dentures, tend to have the highest individual carbon footprints. Nevertheless, although more frequent procedures, such as intraoral examinations and polishing, have a lower individual carbon footprint, their cumulative impact on overall emissions is considerable due to their high frequency (30). This finding has been corroborated by an NHS study (30).

The utilisation of nitrous oxide (N₂O) in dental procedures has also been identified as a significant contributor to CO₂ emissions, resulting in the highest individual carbon footprint among procedures (31).

Dental practices are a significant consumer of various resources, including substantial quantities of energy and water. In England, the average dental practice consumes approximately 33,000 L of water per year, which represents a growing environmental concern in light of the increasing scarcity of this resource (32). While water treatment represents a mere 0.09% of carbon emissions, energy consumption – comprising 7.7% from electricity and 7.6% from gas – exerts a more substantial influence on emissions within the NHS (31).

### 3.6 Waste management in dental practice

#### 3.6.1 Hazardous waste management

The World Health Organisation (WHO) estimates that approximately 85% of healthcare waste is “non-hazardous”, a classification similar to that applied to household waste. The remaining 15% is classified as “hazardous”, comprising 10% infectious waste and 5% chemical or radioactive waste (33). A 2015 WHO report revealed that 58% of healthcare facilities in 24 countries lacked adequate waste management systems, raising concerns about potential safety and environmental risks.

The improper disposal of infectious materials, including needles and blades, presents a significant risk to healthcare workers, the general public, and the environment. This practice contributes to the spread of disease, toxicity, and the emergence of drug-resistant microorganisms (33).

The WHO has established a set of guidelines for the safe management of hazardous waste.

- Infectious waste: Items that have been contaminated with blood or other body fluids must be placed in yellow bags or containers that have been appropriately marked with the biohazard symbol.
- Sharps waste: Instruments such as needles or scalpels must be discarded in yellow receptacles bearing the biohazard symbol and labelled “sharps”.
- Pathological waste: Such waste should be disposed of in containers bearing the biohazard symbol in accordance with the relevant regulations.
- Chemical or pharmaceutical waste: Items such as medicines or x-ray developers should be placed in brown containers marked with the biohazard symbol.

It is imperative that current and future dental professionals are educated on sustainable and safe waste management practices in order to reduce the impact of hazardous waste. The integration of such educational content into the curriculum for dental students may facilitate the promotion of enhanced practices in the future (34).

#### 3.6.2 Management of waste containing heavy metals

##### 3.6.2.1 Conventional x-rays

The utilisation of fixative solutions containing silver thiosulfate in conventional x-ray procedures has the potential to impart significant environmental and health risks if the appropriate disposal protocols are not adhered to. Such risks include adverse effects on the reproductive, respiratory and nervous systems (12). In order to mitigate these risks and prevent contamination of water and soil, the following management measures are recommended (34, 35):

- Fixative Solutions: It is inadvisable to dispose of these solutions via sink drains. Consequently, the aforementioned solutions must be collected and conveyed to an accredited biomedical waste management company, which is equipped to recycle the solutions and recover silver ions for reuse (12, 34).
- x-ray Films: It is inadvisable to dispose of both used and unused x-ray films with general waste. It is recommended that these films be returned to the supplier for recycling in order to prevent environmental contamination. Nevertheless, only approximately five percent of x-ray films are returned for proper disposal (12).
- Digital x-ray Films: The transition to digital x-ray technology provides a safer and more efficient alternative to conventional x-rays. The utilisation of digital systems obviates the necessity for chemical processing, thereby circumventing the disposal issues associated with heavy metals and hazardous chemicals (34, 35).

##### 3.6.2.2 Dental amalgam

The utilisation of dental amalgam gives rise to considerable environmental concerns, primarily due to its mercury content. In response to these concerns, several European countries, including Norway, Sweden and Denmark, enacted legislation banning the use of dental amalgam between 2008 and 2009. Similarly, other countries, including Germany, Finland, the Netherlands, Italy, Spain, and Austria, implemented limitations on the utilization of dental amalgam during this period (14). Since 2018, the European Union has prohibited the use of dental amalgam for the treatment of children under 15 years old and for pregnant or breastfeeding women, unless deemed strictly necessary. The utilisation of dental amalgam is scheduled to be phased out for all patients by 1 January 2025. A prohibition on its manufacture and import into the European Union is set to come into force on 1 July 2026, with the exception of specific medical circumstances (36).

In recognition of the necessity for a global strategy, the United Nations Environment Programme (UNEP) established international regulations with the objective of managing and reducing mercury emissions, including those from dental amalgam. This initiative resulted in the adoption of the Minamata Convention on Mercury in 2013, which was signed by 128 countries and aims to phase out and control the release of mercury into the environment (33).

The principal tenets of the Minamata Convention are as follows:

- Prohibition of predosed capsules: The utilisation of dental amalgam in predosed capsules is explicitly proscribed.
- Prohibition of bulk mercury: The utilisation of bulk mercury is expressly forbidden.
- Amalgam separators: The mandatory installation of amalgam separators with a minimum retention rate of 95% is required in order to effectively capture and retain mercury particles.
- Certified disposal: The collection and disposal of amalgam waste must be conducted by certified waste management companies in order to guarantee the implementation of appropriate management procedures.
- Restrictions on use: The utilisation of dental amalgam is proscribed in the case of primary teeth, in patients under the age of 15, and in pregnant or lactating women, unless it is determined to be indispensable.

The introduction of amalgam separators and filters has been demonstrated to be an efficacious intervention, with a reduction in mercury levels in wastewater of approximately 90% (14). Furthermore, the introduction of selenium filters in crematorium stacks has been demonstrated to effectively mitigate mercury emissions from the cremation of individuals with amalgam restorations (14).

### 3.7 Energy efficiency and sustainable water management in dentistry

It is estimated that the energy consumption related to buildings accounts for approximately 15% of the carbon footprint of primary dental care (37). The reduction of energy consumption has the dual benefit of reducing the environmental impact and providing economic advantages to dental practices. Strategies to enhance energy efficiency encompass the integration of energy-efficient equipment and systems, in addition to the deployment of LED lighting and energy-saving monitors. The installation of motion sensors for lighting can result in a notable reduction in energy consumption in unoccupied rooms. Similarly, the utilisation of natural light can assist with regulating both lighting and temperature. The utilisation of blinds can assist in the regulation of solar heat gain, thereby facilitating an enhanced optimisation of energy utilisation. Furthermore, the installation of solar panels represents a viable option for harnessing renewable energy sources (34). It is of the utmost importance to ensure that equipment is properly maintained on a regular basis in order to guarantee both its longevity and optimal efficiency (38).

The average dental practice utilises approximately 259,000 L of water per annum (36). A number of measures can be implemented in order to reduce water consumption. The installation of automatic taps can assist in limiting water flow, while the utilisation of water-efficient equipment designed to operate with minimal water consumption is also advantageous. The utilisation of dry vacuum systems can additionally contribute to a reduction in water consumption. The monitoring of water usage through the deployment of water meters facilitates the identification of instances of excessive consumption, thereby enabling the implementation of corrective measures (38).

The promotion of responsible water use has the potential to augment the efficacy of water conservation initiatives. The collection and utilisation of rainwater for irrigation or non-potable purposes represents a pragmatic strategy for the reuse of water. Furthermore, it is essential to ensure that autoclaves and washing machines are only operated when they are fully loaded in order to achieve optimal efficiency. Furthermore, the selection of cleaning products with a low water consumption rate can contribute to a reduction in overall water usage (38).

### 3.8 Reduce, reuse, recycle, and rethink in dentistry

The 4Rs (reduce, reuse, recycle, and retrain) provide a framework for minimizing the environmental footprint of dental practices. These principles address the issues of resource consumption, waste generation and the overall sustainability of dental care.

#### 3.8.1 Reducing waste and CO_2_ emissions in dental practice

The minimisation of waste and CO₂ emissions in dental practices can be effectively achieved through several approaches that focus primarily on the prevention of oral disease and the provision of high-quality care (39).

The key measures for effective reduction, as outlined by Martin and Mulligan, focus on several essential aspects of dental practice (40). Preventive care entails the implementation of a comprehensive assessment and management strategy that addresses both local and systemic risk factors. A patient-centred preventive approach has the potential to significantly reduce the necessity for more extensive treatment in the future (39). In the context of surgical care, the enhancement of clinical efficiency necessitates the integration of knowledge, skills and experiential learning with effective teamwork. This optimisation enables procedures to be conducted in an efficient manner, which in turn reduces waste and resource consumption. In addition, integrated care emphasises the role of the dentist and the dental team in taking the lead in promoting good clinical practice, focusing on the development of structured treatment plans that actively involve patients in their care (40). This approach ensures that treatments are carefully planned and targeted, reducing the need for unnecessary interventions and the waste associated with them. Ultimately, promoting patient ownership of their care is critical to developing a culture of excellence. By encouraging active participation in continuing professional development, practitioners can demonstrate their commitment to continuous improvement, ultimately enhancing the quality of care delivered (40).

By concentrating on these strategies, dental practices can markedly diminish waste and CO₂ emissions while enhancing patient care and outcomes.

In conclusion, attending dental appointments at intervals tailored to an individual's disease risk level is essential for effective disease prevention and minimizing the need for extensive material use and multiple treatments, while also reducing unnecessary patient journeys that significantly contribute to the carbon footprint of dentistry (39, 41). To illustrate, although the carbon footprint of fluoride varnish application is relatively high, this preventive intervention can prevent the development of carious lesions, thereby reducing the necessity for restorative treatments over time, which would otherwise generate even greater carbon emissions (38). In instances where oral healthcare is required, it is incumbent upon dentists to prioritise the utilisation of high-quality materials and to perform procedures in a competent manner, thereby ensuring the durability and efficacy of the treatments provided (39, 41).

In order to further reduce CO₂ emissions, it is recommended that both patients and staff utilise sustainable transport options, including walking, cycling, carpooling, the use of electric vehicles and car-sharing services (10, 42). Additionally, dental practices should be situated in areas with convenient access to public transportation, dedicated bicycle lanes, and electric vehicle charging infrastructure to facilitate the utilisation of sustainable transportation options (10, 42). Moreover, walking and cycling confer not only environmental benefits but also economic and cardiovascular health benefits (10).

The effective management of appointments is a fundamental aspect of any healthcare system. Strategies may include scheduling appointments for multiple family members on the same day, increasing the number of procedures per visit, or determining appointment intervals on the basis of the patient's caries index or risk of developing oral pathology (10).

The advent of the SARS-CoV-2 pandemic has precipitated the integration of telemedicine into the practice of dentistry (41). The utilisation of digital platforms, such as oral cavity photography and video consultations, enables the issuance of prescriptions, the formulation of treatment plans and the monitoring of postoperative recovery or disease progression without the necessity for physical visits. This approach effectively reduces the frequency of patient visits, thereby minimising carbon emissions and waste generation (41). Furthermore, online communication facilitates remote lectures and case discussions between colleagues situated in disparate geographical locations (10).

Notwithstanding the advantages associated with its use, telemedicine is not yet a universal practice among patients and dentists. The utilisation of telemedicine is impeded by a number of factors, including a lack of familiarity with the requisite technologies, restricted internet access, communication difficulties and the inherent limitations of diagnosing conditions based on two-dimensional images alone (43).

In conclusion, the reduction of the environmental impact of dentistry necessitates the minimisation of waste generation. Effective product management is of critical importance, including the utilisation of items prior to their expiration date and the avoidance of the acquisition of surplus stock (32). It is recommended that, where feasible, digital formats be selected in preference to paper in order to minimise further environmental impact (32).

#### 3.8.2 Reuse

In clinical settings, the reuse of materials is often subject to restrictions due to concerns about contamination and cross-infection. Nevertheless, there are numerous methods for the effective implementation of reusable items. To illustrate, the utilisation of washable cloth gowns in lieu of disposable alternatives can diminish the generation of waste. Similar methodologies can be employed with regard to bibs and operating tables constructed from reusable materials (44).

Furthermore, the selection of glass or stainless-steel beakers in lieu of disposable paper or plastic alternatives can also facilitate sustainability. The utilisation of perforated metal boxes for the sterilisation of instruments represents an efficacious strategy, as it obviates the necessity for individual sleeves (44).

Furthermore, the option of autoclavable or stainless-steel saliva aspirators permits reuse following the requisite sterilisation process. Furthermore, the utilisation of stainless steel impression trays can supplant the necessity for disposable plastic trays. Additionally, the employment of glass or autoclavable syringes for irrigation is recommended. Furthermore, the selection of autoclavable irrigation tips can contribute to a reduction in waste (31, 38, 44).

#### 3.8.3 Recycle

As indicated by data from the European Parliament, the proportion of plastic waste recycled within the European Union in 2018 was 25% (44). In the field of dentistry, the recycling of plastics is of paramount importance, given their significant environmental impact. The effective recycling of plastic in clinical settings necessitates the involvement of a number of stakeholders, including dentists, manufacturers, and waste collection and processing companies (11).

It is imperative that a number of pivotal measures be implemented in order to address the concerns pertaining to the recycling of medical plastics. Firstly, it is imperative that dentists are educated about the importance of recycling in order to promote a culture of sustainability within the profession. Furthermore, effective communication with recycling companies is required to clarify that not all plastics generated in medical settings are contaminated and can therefore be safely recycled. Training healthcare professionals in the appropriate methods of plastic separation can serve to enhance the efficacy of recycling initiatives. Furthermore, the reuse of plastics should be encouraged wherever feasible, as this will assist in the reduction of waste (45).

In order to promote a circular economy for plastics in healthcare, it is essential to investigate novel recycling methods and consider the introduction of biodegradable plastics, such as latex gloves, in clinical settings (41, 46). It is of paramount importance to prevent cross-contamination between uncontaminated and contaminated plastics during dental procedures by ensuring the effective organisation of the worktable. The appropriate segregation of materials, such as the differentiation between plastic and paper from sterilisation sleeves, enables the accurate recycling of materials and has the potential to reduce waste by approximately 5 kg per week (38). It is recommended that dental practices give priority to the separation of recyclable waste, including paper, metal, glass and organic waste, and ensure the availability of appropriate recycling bins to support this process (32).

#### 3.8.4 Rethink

The final component of the sustainability framework, designated as “rethinking”, underscores the necessity for a comprehensive reassessment of strategies that can effectively minimise the environmental impacts associated with oral health practices. This involves the integration of principles related to recycling and reduction, in addition to the utilisation of Life Cycle Assessment (LCA) to evaluate and compare the environmental benefits of reusable items, such as those that are autoclaved, in contrast to disposable alternatives. The integration of these principles within the overarching sustainability framework facilitates a more comprehensive understanding of the long-term environmental impacts associated with diverse dental practices (11, 47).

It is imperative that sustainable practices are not confined to consumer behaviour, but rather, are implemented across the entirety of the healthcare supply chain, encompassing production, distribution and delivery (38). In order to promote this holistic approach, a number of strategic measures can be implemented.

First and foremost, transparency is of paramount importance. It is incumbent upon manufacturers to provide clear and comprehensive information regarding the materials and methods employed in the development of their products. The implementation of an environmental rating system could enhance transparency by indicating the sustainability of various products, thereby assisting dental practices in making well-informed purchasing decisions (39, 42). Secondly, it is imperative that comprehensive training programmes are made available to all stakeholders within the supply chain, with the aim of promoting the adoption of sustainability practices that are relevant to their respective roles (48). Furthermore, the formation of research collaborations with the objective of examining sustainability concerns is of paramount importance. Such collaborative endeavours can assist in the identification of current knowledge gaps and the development of targeted strategies to address these challenges (7).

Furthermore, it is imperative that environmental sustainability concepts be incorporated into the curriculum for those pursuing a career in dentistry. Such integration will ensure that future dental professionals are well versed in sustainable practices and understand the role of preventive care in promoting sustainability (7).

It is imperative that legislation and policies that encourage sustainable practices are developed. Such policies could encompass the implementation of incentives to encourage sustainable practices and the advancement of oral health through initiatives such as screening programmes, public awareness campaigns and the fluoridation of water supplies in regions devoid of natural fluoride (7).

## 4 Sustainable alternatives for dental materials

### 4.1 General practitioners

#### 4.1.1 Sustainable oral hygiene measures

The concept of sustainable oral hygiene encompasses not only clinical procedures but also encompasses daily routines and product choices. It is imperative that patients are educated about the environmental impact of their oral hygiene habits. For instance, patients should be counselled to turn off the tap while brushing their teeth in order to conserve water (38).

#### 4.1.2 Dental products

A number of recent studies have assessed the sustainability of a range of dental products, with a particular focus on toothbrushes. A comparative study of the environmental impact of conventional plastic, bamboo, bioplastic with removable heads and electric toothbrushes revealed that electric toothbrushes had the highest carbon footprint (49). Notwithstanding their greater environmental impact, electric toothbrushes have been demonstrated to be more effective than manual toothbrushes at reducing plaque and gingivitis (38, 49). Conversely, bamboo and bioplastic toothbrushes with removable heads have the lowest environmental impact, rendering them more sustainable alternatives to conventional plastic and electric toothbrushes (42). It would be beneficial for future research to investigate the clinical effectiveness of electric toothbrushes in comparison to manual options and to assess the effectiveness of sustainable alternatives that are currently available on the market (32).

#### 4.1.3 Toothpaste

The emergence of sustainable alternatives to traditional toothpaste tubes is attracting increasing attention. A study conducted in Thailand compared the environmental impact of toothpaste tablets (0.7 g per tablet) with that of traditional tube toothpaste (0.25 g to 0.4 g per use) (48). The findings demonstrate that toothpaste tablets have a more pronounced environmental impact than conventional tube toothpaste, particularly in the areas of raw material acquisition, production, transportation, and utilisation. However, the environmental impact of toothpaste tablets was found to be lower during the disposal phase. Furthermore, the composition of tablet toothpaste gave rise to concerns regarding its potential impact on health, given the presence of potentially harmful ingredients such as sugar alcohols, povidone and magnesium stearate. A reduction in the size of the tablet to 0.4 g resulted in a notable decrease in the environmental impact, indicating that smaller tablets may offer a more sustainable alternative (44). Therefore, while toothpaste tablets present certain environmental advantages, their overall impact is contingent upon formulation and size. Consequently, further optimisation is necessary to enhance sustainability and safety.

#### 4.1.4 Dental floss and interdental brushes

A comparative study of various types of dental floss and interdental brushes was conducted to evaluate the sustainability of conventional, toothpick, sponge, and bamboo floss, as well as conventional, toothpick, removable head, and bamboo interdental brushes (50). The findings revealed that toothpick floss exhibited the highest environmental impact among the evaluated types, whereas the environmental impact of other floss options was comparable, with minimal differences. With regard to interdental brushes, those featuring removable plastic heads and bamboo heads exhibited the lowest environmental impact, with bamboo brushes demonstrating the lowest overall footprint (50).

### 4.2 Implantology

From an environmental standpoint, it is of paramount importance that dental professionals, particularly those specialising in implantology, are adequately informed about the sustainability of different implant materials. Such awareness will enable dental professionals to make environmentally responsible decisions without compromising clinical efficacy.

The current trend in implantology is towards the use of ceramic materials in preference to metallic alternatives, motivated primarily by considerations of aesthetics and biocompatibility (51). The objective of a study published in 2019 was to evaluate and compare the mechanical properties of metallic and ceramic materials employed in implantology, as well as their environmental properties, including carbon footprint and resource consumption during production. The materials evaluated in this research included alumina (Al₂O₃), yttria-stabilised polycrystalline tetragonal zirconia (Y-TZP), 316l stainless steel, cobalt-chromium alloy (CoCr), commercially pure titanium (cp-Ti) and alpha-beta titanium alloy (Ti6Al4V) (51). This study concentrated on three mechanical properties: flexural strength, Young's modulus and Vickers hardness. The findings indicated that zirconia provides an optimal equilibrium between these mechanical properties and a reduction in environmental impact. In particular, the manufacturing processes for ceramic materials, such as alumina and zirconia, necessitate less water and energy and result in diminished carbon emissions in comparison to their metallic counterparts (51).

In conclusion, the preference for ceramic materials over metallic materials in implantology has notable environmental advantages. Nevertheless, further research is required to evaluate additional variables and materials employed in this field in order to gain a comprehensive understanding of their environmental impact (51).

### 4.3 Restorative dentistry

As previously stated, resin-based composites are frequently proposed as potential alternatives to dental amalgam for direct restorations. However, these materials may potentially pose environmental and health risks due to the release of microplastics and monomers into the environment (38). Several strategies can be employed to mitigate the contamination of water sources and soil from resin monomers (52).

The initial step is to enhance the polymerisation efficiency of these materials, which will result in a higher conversion rate of monomers into polymers. This can be accomplished by optimising the polymerisation process. Furthermore, the utilisation of rubber dams is advised, particularly during the replacement of composite resin restorations, as this technique can markedly diminish the concentration of monomers in saliva (52).

The application of a glycerine gel represents an efficacious measure, as it facilitates the management of the oxygen inhibition layer on the restoration surface. This results in a reduction of unpolymerised monomers, thereby limiting their release during the polishing process. Furthermore, clinicians must exercise caution to avoid excessive application of composite resin, as this can result in the release of microparticles upon removal (52).

Future research should prioritise the development of resins with higher conversion rates, the exploration of alternative materials, and the creation of specialised equipment designed to filter particles released during the use of these materials, in order to address pollution stemming from resin-based composites (52).

### 4.4 Endodontics

Endodontic treatment makes use of a variety of materials, many of which are designed for single use or have a limited number of applications. These procedures typically consume a considerable amount of energy and water. Due to their inherent complexity, they can be time-consuming and may necessitate multiple sessions, resulting in an increased generation of waste (47).

Endodontic files are typically categorised as reusable instruments; however, their utilisation is constrained by the potential risk of breakage, which limits their application to a specified number of instances. In some countries, such as the United Kingdom, these files are classified as single-use due to the inherent risks associated with their use (47). It is crucial to acknowledge that the majority of endodontic file systems necessitate the utilisation of multiple files throughout the course of treatment. In order to reduce the waste associated with these materials, it would be prudent to explore the use of systems that require fewer files or to consider single-file systems, provided that such approaches do not compromise the efficacy of the treatment (47).

Another strategy to reduce the environmental impact of endodontic procedures is to prioritise single-session treatments whenever feasible. This approach has the additional benefit of reducing waste and minimising emissions associated with patient transport, while also reducing material costs for dental practices (45). Moreover, when clinically appropriate, the utilisation of vital pulp therapy can also confer environmental benefits, as this technique typically generates less waste in comparison to conventional endodontic treatments (47, 53).

### 4.5 Fixed and removable prosthodontics

The average number of patient visits for removable and fixed dentures is approximately five, with the exact figure dependent on the complexity and type of prosthesis. Furthermore, this process results in increased carbon emissions associated with patient travel and the generation of significant waste due to the extensive use of materials. Furthermore, the environmental impact of prosthetists’ laboratory work must be taken into account (54).

A number of strategies can be employed in order to reduce the environmental impact of this area. The initial step is the utilisation of sophisticated technologies, including intraoral scanners and computer-aided design and manufacturing (CAD/CAM) systems, which can markedly diminish the environmental impact. Despite the aforementioned limitations, these technologies contribute to a more efficient digital workflow by reducing chair time and material waste (54). Intraoral scanners occasionally fail to provide accurate full-arch readings, while CAD/CAM systems generate pollution and consume significant amounts of energy.

Secondly, the introduction of innovative materials and methods provides further opportunities for environmental improvement. To illustrate, the creation of reusable tips comprising a double-helix structure and a detachable plastic cover enables the removal of surplus material and permits repeated utilisation of the tips (48). Furthermore, ongoing research into the recycling of zirconia for reuse in prosthesis manufacture has demonstrated the potential to reduce reliance on virgin ceramic materials, thereby minimising overall waste (38).

The integration of advanced technologies and innovative materials within dental practices can result in a notable reduction in environmental impact, while simultaneously maintaining the highest standards of patient care.

### 4.6 Orthodontics

The implementation of sustainable practices in orthodontics necessitates the selection of environmentally friendly products and the adoption of conscientious methodologies. A variety of strategies can be employed to reduce the environmental impact of orthodontic procedures.

One such approach is the utilisation of biodegradable packaging. This is exemplified by the provision of orthodontic kits with a greater number of brackets in biodegradable containers, which serves to diminish the quantity of plastic waste generated (52). Another efficacious strategy is the sterilisation and reuse of archwires, particularly those employed during the concluding phase of treatment. As an illustration, nickel-titanium (NiTi) archwires can be sterilised in a 2% glutaraldehyde solution for a period of 10 h following a six-week utilisation period, thereby facilitating their reuse and contributing to a reduction in waste (55). The utilisation of self-etching adhesive systems for bracket bonding represents an effective strategy for the reduction of water waste (56).

The utilisation of self-ligating brackets is also advised, as these devices do not necessitate the use of elastomers and therefore have a reduced environmental impact in comparison to conventional brackets (56). In the case of intermaxillary elastics, it is recommended that consideration be given to the use of latex as an alternative to synthetic elastics. Moreover, the reuse of brackets removed during treatment is encouraged. These brackets can be cleaned with an aluminium oxide jet or sandblasted to remove adhesive residues and prepare their surfaces for reattachment (53, 57). Furthermore, the reuse of microimplants within the same patient, following the appropriate sterilisation procedures, is a potential avenue for consideration (47, 58). Additionally, orthodontists may opt to utilise intraoral scanners in lieu of conventional impressions (59).

Despite their popularity, the use of plastic invisible aligners presents a significant environmental challenge. The typical replacement interval for these aligners is one to two weeks, which results in a considerable quantity of plastic waste (60). While some companies have initiated aligner recycling programmes, these efforts are currently limited in scope. Furthermore, the resins used for 3D printing aligner models may pose an environmental risk. Although the investigation of recyclable materials for 3D printing and the improvement of recycling programmes are potential strategies to mitigate these issues (61), it remains speculative whether these efforts will effectively reduce the overall environmental impact.

## 5 Conclusions

The acute effects of climate change are evident in the occurrence of extreme natural phenomena, including wildfires, the melting of ice caps and prolonged droughts. The exacerbation of this global crisis can be attributed, at least in part, to human activities, particularly the destructive exploitation of natural habitats for the purpose of resource extraction. Pollution, particularly that resulting from plastic waste, represents a significant threat to marine biodiversity and disrupts oceanic pH levels, creating a series of cascading effects that undermine ecological balance.

Furthermore, the consequences of climate change extend beyond mere environmental deterioration, with far-reaching ramifications for human well-being. This article elucidates the manner in which systemic alterations in health can be attributed not only to natural disasters, but also to the immediate consequences of clinical practice in disciplines such as dentistry. It is becoming increasingly evident that immediate action is required in this field.

In order to address the environmental footprint of dentistry, it is necessary to implement a unified strategy that aligns with the United Nations Sustainable Development Goals (SDGs), as outlined in the 2030 Agenda and summarised in Table 2. These goals promote the integration of sustainable practices across a range of sectors. The interconnection between the sustainability measures presented here and the selected FDI-endorsed SDGs provides a crucial framework for mitigating the environmental impact of dental practices, thus contributing to the advancement of global sustainability initiatives.

**Table 2:** Correlation of sustainable practices in dentistry with United Nations' Sustainable Development Goals (SDGs).

Sustainable practices	SDG 3	SDG 4	SDG 6	SDG 8	SDG 9	SDG 12	SDG 13	SDG 17
Management of hazardous waste and heavy metals	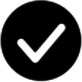	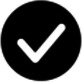	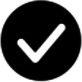		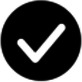			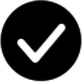
Reduce, reuse, recycle, and rethink	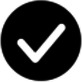	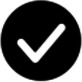		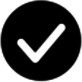	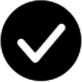	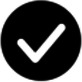	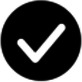	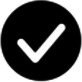
Sustainable dental or oral hygiene materials		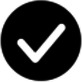	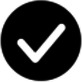		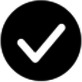	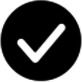	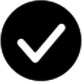	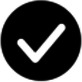
Conscious consumption of energy and water			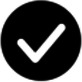		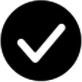	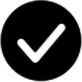		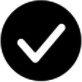

In conclusion, this literature review has identified significant sustainability gaps in the dental sector and highlighted the environmental and health risks associated with current clinical practices. The findings indicate that a range of sustainable strategies can be integrated into dental care in alignment with the UN's 2030 Agenda, with the potential to significantly reduce the sector's environmental footprint. These include pivotal domains such as waste management, clinical protocols, materials selection, and resource consumption.

Nevertheless, despite the potential of these measures to make a meaningful contribution to sustainability, significant challenges remain that require further investigation. It is imperative that further research be conducted in order to enhance our comprehension of these intricate matters and to enhance the precision of sustainable methodologies in the field of dentistry. Furthermore, it is of the utmost importance to educate dental professionals about sustainable practices in order to facilitate their integration into daily clinical practice.

The findings of this review give rise to a number of pressing recommendations. Firstly, the development of educational initiatives aimed at training dental professionals in sustainable practices, with a particular focus on their role in minimising environmental impact, is of paramount importance. Secondly, research should be actively pursued with the objective of identifying and evaluating sustainable materials and technologies in dentistry. This research should assess the effectiveness and environmental benefits of these materials and technologies in comparison to traditional options. Thirdly, advocacy for policy changes that promote sustainability within the dental sector must be aligned with global sustainability goals.

It is also imperative to enhance patient awareness of the environmental impact of oral hygiene practices and the significance of sustainable product selections. Ultimately, the promotion of sustainable practices and products in dentistry will require the establishment of collaborative initiatives between dental associations, manufacturers, and policy makers.

By adopting these recommendations, the dental community can address the urgent issues of climate change while enhancing health outcomes for individuals and communities, thereby paving the way for a genuinely sustainable future for all.
